# Dietary Factors Modulating Colorectal Carcinogenesis

**DOI:** 10.3390/nu13010143

**Published:** 2021-01-03

**Authors:** Filippo Vernia, Salvatore Longo, Gianpiero Stefanelli, Angelo Viscido, Giovanni Latella

**Affiliations:** Department of Life, Health, and Environmental Sciences, Division of Gastroenterology, Hepatology, and Nutrition, University of L’Aquila, Piazza S. Tommasi, 1- Coppito, 67100 L’Aquila, Italy; filippo.vernia1@gmail.com (F.V.); salvator.longo@gmail.com (S.L.); giastefanelli@gmail.com (G.S.); angelo.viscido@univaq.it (A.V.)

**Keywords:** colorectal cancer, diet, nutrition, red meat, processed meat, fiber, vitamins, short chain fatty acids (SCFA)

## Abstract

The development of colorectal cancer, responsible for 9% of cancer-related deaths, is favored by a combination of genetic and environmental factors. The modification of diet and lifestyle may modify the risk of colorectal cancer (CRC) and prevent neoplasia in up to 50% of cases. The Western diet, characterized by a high intake of fat, red meat and processed meat has emerged as an important contributor. Conversely, a high intake of dietary fiber partially counteracts the unfavorable effects of meat through multiple mechanisms, including reduced intestinal transit time and dilution of carcinogenic compounds. Providing antioxidants (e.g., vitamins C and E) and leading to increased intraluminal production of protective fermentation products, like butyrate, represent other beneficial and useful effects of a fiber-rich diet. Protective effects on the risk of developing colorectal cancer have been also advocated for some specific micronutrients like vitamin D, selenium, and calcium. Diet-induced modifications of the gut microbiota modulate colonic epithelial cell homeostasis and carcinogenesis. This can have, under different conditions, opposite effects on the risk of CRC, through the production of mutagenic and carcinogenic agents or, conversely, of protective compounds. The aim of this review is to summarize the most recent evidence on the role of diet as a potential risk factor for the development of colorectal malignancies, as well as providing possible prevention dietary strategies.

## 1. Introduction

Colorectal cancer (CRC) is a highly common malignancy, being the third leading cause of cancer death worldwide. It has been estimated that in 2018 the incidence of new cases has been approximately 2 million, determining 1 million deaths worldwide [1].

CRC is a silent disease, often presenting in advanced form, developing as a slow, multi-step process, that takes approximately 5–10 years from premalignant lesions to CRC [2].

Moreover, being influenced by several risk factors related to the patient (including age, sex, and familial predisposition) and environment (diet, excess body weight, and tobacco use), CRC may be modulated by targeted risk reduction measures [3].

Although in the past years the carcinogenic effect of diet was mainly attributed to high-fat, high-calorie diets, an increasing amount of attention is now focused on the specific role of different nutrients like fibers, vitamins, and minerals, as well as of intestinal microbiome metabolism.

Indeed, the World Cancer Research Fund/American Institute of Cancer Research (WCRF/AICR), suggests that CRC could be prevented in up to 50% of cases, modifying risk factors such as diet and lifestyle behaviors [4]. The same report states that consuming whole grains, dietary fiber, and dairy products decreases the risk of CRC. Conversely, the Western diet, characterized by a high intake of red and processed meat (rich in heme iron) and fat increases the risk of CRC. Some evidence supports the protective effect of vitamin C, fish, and vitamin D, in decreasing the risk of CRC [4].

Thus, dietary intervention has emerged in the last decades as an attractive strategy to reduce the occurrence and progression of CRC.

The aim of this narrative review is to summarize the most recent evidence on the role of diet as a potential risk factor for the development of colorectal malignancies, as well as providing dietary strategies that may counteract this effect.

## 2. Materials and Methods

A systematic electronic search of the English literature up to November 2020 was performed using Medline, Excerpta Medica database (EMBASE), Web of Science, Scopus, and the Cochrane Library. The search strategy used a combination of Medical Subject Headings (MeSH) and keywords as follows: “colorectal cancer”, “CRC”, “colorectal adenoma”, “risk factors”, “carcinogens”, “diet”, “dietary habit”, “food”, “meat”, “red meat”, “processed meat”, “heme iron”, “proteins”, “fat”, “lipids”, “carbohydrates”, “sugar”, “refined sugars”, “nitrates”, “nitrites”, “nitrosamines” “dietary fibers”, “vegetables”, “fruit”, ”dietary minerals”, “calcium”, “selenium”, “nutrients”, “vitamins”, “vitamin C”, “vitamin D”, vitamin E”, “antioxidants”, “gut microbiota”, “fecal organic anions”, “fecal short-chain fatty acids”, “short chain fatty acids”, “SCFA”, “butyrate”.

Three authors (Filippo Vernia, Salvatore Longo, and Gianpiero Stefanelli) identified relevant articles by screening the abstracts. Additional studies were selected after a manual review of the reference list of the identified studies and review articles. Any discrepancy was resolved by consensus, referring to the original articles. Out of 4573 citations, 118 relevant articles were selected and included in the present narrative review.

## 3. Red and Processed Meat and Colorectal Cancer

Processed meat, defined as meat that has been transformed through salting, curing, fermentation, smoking, or other processes to enhance flavor or improve preservation, and red meat have been included in 2015 in the list of substances that can contribute to the development of cancer, by the International Agency for Research on Cancer (IARC). Based on available data, the study group concluded that processed meat is carcinogenic (Group 1), while red meat is probably carcinogenic (Group 2A) [4].

Therefore, it has been recommended that the intake of red meat is limited to less than three portions weekly, corresponding to 350–500 g (12–18 oz) of cooked weight [4]. Processed meat, more so smoked and nitrite-containing foods, should be avoided, as no level of intake can confidently be associated with a lack of risk [4].

The association between CRC and enhanced consumption of red and processed meat, leading to this recommendation, is supported by considerable evidence.

The European Prospective Investigation into Cancer and Nutrition (EPIC) provided strong evidence of this association in more than 500,000 individuals [5] as habitual meat consumers had a 20% higher risk to develop CRC, compared to non-consumers or occasional consumers [5].

Similarly, the Norwegian Women and Cancer (NOWAC) cohort, including 88,000 women, concluded that consuming more than 60 g processed meat a day doubles the risk of developing CRC compared to less than 15 g [6].

Other cohorts reported that the daily ingestion of 100 g of fresh red meat determines a 17% increased risk of CRC, while 50 g processed red meat raises the risk by 18% [4].

In contrast, there is not enough evidence to support the role of white meat consumption in increasing the risk of CRC.

The carcinogenic effects of red and processed meat are mainly related to the presence of growth-promoting dietary components, such as heme and arginine, enhanced mutagenic intestinal environment, and intestinal inflammatory response [7].

The best-studied mechanism involves heme iron [8], converted in the colon into cytotoxic heme factor (CHF). This damages the surface epithelial cells [9] and induces reactive epithelial hyperproliferation. The abundance of mucin-degrading bacteria, such as *Akkermansia muciniphila*, and sulfate-reducing bacteria enhances these effects [9].

Colonic damage is boosted by the production of reactive oxygen species (ROS) induced by heme iron, which favors the oxidation of DNA, lipids, and proteins [10].

Heme has been shown in animal models to inhibit colonocyte apoptosis and exfoliation, providing an additional mechanism contributing to carcinogenesis [11].

Heme iron increases the production of N-nitroso compounds (NOCs) [12]. Nitrosamines, synthesized by the intestinal microbiota from the nitrites, are particularly active carcinogenic compounds [13]. Processed meat is still often supplemented with nitrites to favor preservation. Free nitrosyl heme has been shown in animal models to synthesize more NOCs during cooking than native heme [14]. Moreover, hemoglobin and myoglobin directly react with nitrites, forming N-nitroso-hemoglobin and N-nitroso-myoglobin, possibly explaining the dose-dependent effect of red meat [15].

Additionally, arginine, as a precursor of polyamines, has been proposed as a potential CRC risk factor [16]. Polyamines, such as putrescin, spermidine, and spermine, are involved in cellular processes, including proliferation, and provide an additional mechanism linking red meat to CRC [7,17].

Several genotoxic and mutagenic substances particularly NOCs and oxidized lipids result from preservation, curing, and/or cooking process and bacterial metabolic activity [7,18]. Heterocyclic amines (HCAs) formed upon over-heating amino acids and sugars, either alone or in association with polycyclic aromatic hydrocarbons (PAHs), and nitrites/nitrates, are also harmful [7].

An additional mechanism contributing to CRC is lipidic peroxidation [7]. This results in the production of O6-carboxymethyl guanine adducts and other molecules with toxic and mutagenic effects [19]. Interestingly, lipidic peroxidation is further enhanced by heme iron [20], in relation to the catalytic activity on the bacterial production of aldehydes, which in turn increases the genotoxic effect [21].

Some evidence supports the potentially harmful effect of other protein fermentation products. Hydrogen sulfide promotes both inflammation and the proliferation of CRC cells [22,23], 4-hydroxyphenyl-acetic acid is genotoxic, and phenylacetic acid and phenol exert cytotoxic effects [24].

## 4. High-Fat Diet, Biliary Acids, and Colorectal Cancer

Recent data further support the tumor-promoting activity of high-fat diets, largely depending on the complex interactions between the gut microbiota and bile acid metabolism [25,26].

Excess dietary fat stimulates the hepatic synthesis of bile acids, resulting in increased amounts of bile acids escaping the ileal reabsorption by apical sodium-dependent bile acid transporter or the ileal bile acid transporter. As a consequence of their deconjugation by microbial enzymes, the ratio of primary to secondary bile acids entering enterohepatic cycling and within the colonic lumen is modified [26].

Recent data support the well-known concept that lower levels of bile acids and 7αdehydroxylating bacteria are present in the stool of healthy rural Africans than in those of healthy African Americans. The latter group consuming a high-fat, low-fiber diet has a much high prevalence of CRC, compared to the former one, consuming a low-fat, high-fiber diet [27]. Interestingly, a diet switch leads to lower fecal levels of bile acids and 7α-dehydroxylating bacteria in African Americans, while the opposite is true for rural Africans, in parallel to an increase of mucosal markers associated with CRC risk [28].

Despite several studies aiming to clarify the tumor-promoting function of bile acids, including oxidative stress and inflammation, the underlying molecular mechanisms remain unclear. Multiple mechanisms stimulating CRC cell proliferation have been advocated for secondary bile acids, including receptor-dependent signaling pathways [29], the activation of β-catenin cell-signaling, extracellular signal-regulated kinases 1 and 2 (ERK1/2), signaling via activator protein 1 (AP1) and c-Myelocytomatosis (c-Myc) target pathways [30,31]. An additional CRC pathway activated by secondary bile acids is the nuclear factor kappa B (NF-κB) pathway [32].

Recently, the role of farnesoid X receptor (FXR) signaling in gut—liver crosstalk has been identified as central in the control of intestinal epithelial cell proliferation. FXR deficiency promotes the proliferation of colonic epithelial cells accompanied by a high expression of cyclin D1 [33] in keeping with the reduced expression of FXR in precancerous lesions and CRC [34]. Animal studies show that a high-fat diet induces an altered activity of FXR, correlating with higher numbers of Ki-67+ cells in colonic crypts [35]. The cell cycle antigen Ki-67 is a nuclear protein associated with cellular proliferation.

## 5. Fibers and Colorectal Cancer

The observation that the prevalence of CRC increases inversely to the intake of dietary fiber led in the last decades to extensive investigation on the protective role of fibers, especially whole grain [4,36]. Similarly, the European Prospective Investigation into Cancer and Nutrition (EPIC) study documented a 40% reduction in CRC risk in the highest quintile of fiber intake compared with the lowest [5].

This has also been recently confirmed by an update of the National Institutes of Health and American Association of Retired Persons (NIH-AARP) Diet and Health Study [37], following more than 10,000 participants over 15 years. The study confirms that the intake of whole grains, but not dietary fiber from other origins, is inversely associated with CRC risk. Participants in the highest quintile of intake of whole grains had a 16% lower risk of CRC compared with those in the lowest quintile [37]. This study also strongly suggests that the protective effect depends on the whole grain-containing food, in which other constituents (e.g., folate) are present, more than the fiber content. This important point was previously undefined [38].

The chemoprotective effect of fibers in different colonic segments however seems to vary with food source [39]. Similar partially conflicting conclusions have also been drawn for the inverse association between fibers and adenoma [40,41].

Several mechanisms have been suggested to explain how dietary fiber may reduce CRC, ranging from dilution of carcinogens in larger amounts of stool resulting from the ingestion of non-fermentable fiber, to highly sophisticated intracellular metabolic effects triggered by fermentation by products [36]. The mere reduction of fecal pH induced by dietary fiber fermentation decreases the production of bacterial carcinogens deriving from bile acid metabolism [42]. Intraluminal acidification reduces intestinal transit time and colonocyte exposure to carcinogens, thus representing an additional chemoprotective effect of dietary fiber [43]. Moreover, it has been reported a higher large bowel intraluminal pH in patients with CRC compared to healthy controls [44,45], more so when measuring the pH of the colonic mucosal surface than that of luminal contents [46].

Fiber fermentation by gut microbiota leads to the production of short-chain fatty acids (SCFA), predominantly acetate, butyrate, and propionate, in a ratio of 3:1:1 [47].

Butyrate, besides representing the main energy source in normal colonocytes, shows a protective effect on colonic mucosa [48]. Butyrate has anti-inflammatory properties, as it has been reported a reduction of plasmatic pro-inflammatory cytokines and an increment of regulatory T-lymphocytes in animal models [49].

Butyrate reduces proliferation and increases differentiation of CRC cells [48].

Furthermore, it has been also reported that butyrate induces apoptosis in CRC cells [50], acting as a potent histone deacetylase inhibitor and through the activation of the Fas receptor-mediated extrinsic death pathway [51].

An additional mechanism by which butyrate may determine a protective effect against CRC is the induction of colonocyte apoptosis, due to the production of cellular reactive oxygen species (ROS) [52], which determines the release of proapoptotic factors [53].

Of particular interest is the differing behavior of butyrate on normal colonic cell lines and colon cancer cells. In normal cells, it increases cell weight, increases DNA content, and increases proliferation and crypt length, with the energy provided. This is in line with the mucosal anti-inflammatory effect of butyrate reported in ulcerative colitis [54].

In cancer cells favors the arrest in the G1 phase and differentiation due to a direct effect on mutated G protein and decreases cloning efficiency and adhesion to laminin [55]. The reduction of cellular proliferation and increase of differentiation of CRC cells has also been described in small in vivo studies [56].

Other putative beneficial mechanisms of butyrate are represented by the modulation of micro-RNAs (miRNAs), small non-coding RNA molecules. High butyrate levels reduce the expression of MYC oncogene, which in turn reduces the levels of the miRNA-17-92 cluster miRNAs, playing a central role in cellular proliferation, metastasis, and angiogenesis [57].

More recently attention has been paid to the differing effects of different fibers (e.g., soluble and insoluble) which modulate the composition of the microbiota and influence the microbial production of butyrate and other intermediate compounds of bacterial metabolism [58].

Despite the vast amount of evidence deriving from epidemiologic, basic science, and animal studies, results in humans are still conflicting and largely inconclusive [59,60].

## 6. Vitamins, Minerals, and Colorectal Cancer

Besides the higher intake of fibers associated with a lower intake of meat, consumption of fruit and vegetables may reduce the incidence of CRC [61] in relation to their content of specific micronutrients, such as vitamins and polyphenols [36].

Vitamin C and E showed a direct tumor-suppressing effect on CRC cell lines [62,63]. A negative association has been also reported in a cohort from Shanghai [64] for vitamin C, and in a Canadian cohort [65] for vitamin E, but not in other studies [66]. The possible protective effect of these vitamins in CRC is thus debated although the removal of free radicals counteracts the production of NOCs from nitrites and nitrates [67].

However, several intervention studies did not provide consistent support for an inverse association between supplemental vitamin E or C, and CRC [68,69]

It has been advocated that vitamin D also reduces the risk of CRC inhibiting neo-angiogenesis and cellular proliferation, and inducing apoptosis [70,71]. A metanalysis published in 2005 highlighted the need to identify the required levels to exert this protective effect [72]. The issue is still unsolved, but very high Vitamin D concentrations are likely required. In a recent prospective trial on 25,871 men, however, vitamin D supplementation did not reduce the incidence of invasive cancer after a 5.3-year follow-up [73]. Therefore, these data seem to limit the effectiveness of this intervention.

An inverse association with CRC has been also reported for selenium and calcium.

The putative effect of selenium is due to its antioxidant and anti-inflammatory properties, and upregulation of the glutathione peroxidase 2 [74].

Despite a meta-analysis [75] reported that selenium supplementation is associated with a reduction in the incidence of CRC, recent studies did not confirm the protective effect [76,77].

Similar conclusions may be drawn for calcium intake. An old, pooled analysis from prospective cohorts reported a 22% reduction in CRC incidence in the highest quintile of dietary calcium intake compared to the lowest quintile [78]. More recent studies did not confirm a protective role of this mineral [79,80], however, the evidence may be considered somewhat stronger than for selenium.

Again, the underlying mechanisms are not clear. Calcium possibly acts indirectly, form soaps that bind secondary bile acids and fatty acids [81], as well as directly, through the reduction of cell proliferation and inducing apoptosis [70,71]. It has also been reported that calcium may also contrast the cytotoxic effect of heme iron on the colonic mucosa [82].

Other putative mechanisms involve modulation of the expression of transforming growth factor-alpha and beta 1 [83] and the expression of the b-catenin gene [84].

Among the most relevant microbial metabolites having a protective effect against CRC, strong evidence is reported on niacin. It suppresses inflammation and tumor progression acting both on macrophages and dendritic cells, as well as promoting differentiation of Treg and IL-10-producing T cells [85].

## 7. Gut Microbiota and Colorectal Cancer

The role of gut microbiota in the development of CRC through specific biochemical pathways is becoming increasingly evident [86,87]. Microbes produce toxic metabolites or carcinogenic products [88]. Moreover, other products of bacterial metabolism may exert an indirect effect, as lactate that represents the prevalent energy source in CRC cells [89,90].

Similarly, bacterial toxins such as the enterotoxigenic one produced by *Bacteroides fragilis* play a central role in CRC development through several mechanisms such as the activation of β-catenin signaling, the cleavage of E-cadherin, and the induction of NF-κB pathway [91]. Similar pathways are also activated by *Fusobacterium nucleatum* and *Escherichia coli* [92,93].

However, the relationship between noxious bacterial species and CRC is probably bidirectional, as it has been recently reported that after CRC treatment the gut microbiota changes, becoming more similar to that of patients with a normal colon [94].

Conversely, as previously mentioned, some bacterial metabolites such as SCFA or niacin have protective effects against CRC. Nonetheless, the specific role of most microbial metabolites remains unclear or displays differing effects in different conditions. Succinate, for example, has been shown to inhibit CRC cell proliferation in some studies [95], while others suggest that succinate can promote metastasis [96].

Further complicating the interplay between host and microbiota, most bacterial metabolites are not produced by individual species. End-metabolic compounds deriving from the metabolism of some bacteria, represent intermediate metabolites for others. For example, butyrate is produced by several bacteria distributed across four different phyla: *Firmicutes*, *Fusobacteria*, *Spirochaetes*, and *Bacteroidetes* [97]. At the same time *Roseburia spp*. can use acetate, produced by other bacteria, for synthesizing butyrate [98], while methanogenic *Archaea* and sulfide-producing bacteria instead reduce or further metabolize butyrate in the colon [99]. These interactions show how the production of microbial metabolites by helpful/harmful species may not correspond to the concentration acting on the colonic mucosa, limiting our knowledge on the doses of each bacterial compound needed to have a beneficial/detrimental effect.

Diet may thus affect CRC risk directly providing helpful or noxious substances, modifying the intraluminal media, influencing and modulating the abundance and type of microbial community as well as increasing or reducing the production of specific metabolites [100]. To further confound the issue, this complex interaction is further modulated by specific metabolic characteristics and microbial communities that are different across individuals [101].

Despite the evidence is still conflicting [102,103], recent studies, suggest a possible positive immunomodulatory effect, as well as an improvement of the gut-barrier activity induced by several probiotics [104,105].

**Figure 1:**
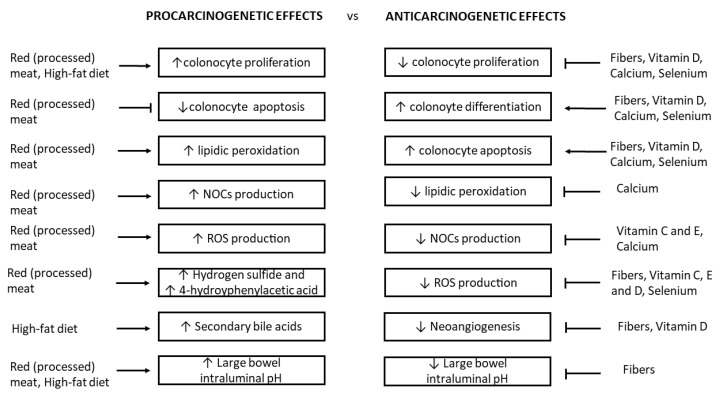
Schematic representation of the mechanisms of specific dietary components in improving or contrasting the carcinogenic processes on large bowel mucosa. ↑: increase; ↓: reduction.

Further studies are therefore needed to clarify whether specific nutrients exert their protective or damaging effect directly on the colorectal epithelium or indirectly by inducing changes in the metabolism of the microbiome (Figure 1).

The rapidly increasing knowledge of the complex crosstalk between the host, gut microbiome, and microbial metabolites suggests that tailored dietary intervention might become pivotal in the prevention of several diseases, including CRC.

## 8. Nutrients, Epigenetics and Colorectal Cancer

In the last decade, an increasing amount of attention has been focused on the connection between gene regulation and CRC. Several nutrients affect gene expression, binding transcription factors, or being involved in post-translational modifications, such as acetylation and methylation.

The promotion of intestinal carcinogenesis may be favored by epigenetic modifications triggered by red and processed- meat.

Just one week of a high red meat diet induces the activation of the Nucleosome Remodeling and Deacetylase (NuRD) complex involved in methylation-mediated gene silencing, in human gut biopsy samples [106].

Heme-supplemented diet suppresses *Wif1* and *BMP2* genes—which antagonize the Wnt signaling cascade and promote differentiation of intestinal cells—in mice [11].

Heme also binds other transcription factors, including Bach1, promoting histone deacetylation, and repressing a subset of *p53* target genes, involved in cellular senescence [107,108]. Mitotic chromosome alignment during metaphase is also affected by Bach1 [109].

Conversely, the Mediterranean diet exerts a protective effect against CRC mediated by DNA methylation of the human runt-related transcription factor 3 (RUNX3) [110]. The methylation of apoptosis-related genes following the administration of other nutrients like *n*-3 polyunsaturated fatty acids and fibers, directly or mediated by SCFA and other fermentation products, is also protective [111,112].

The same proves true for other dietary components, such as polyphenols [113] vitamins [114,115,116], and minerals [116,117] alone or in combination.

Dietary bioactive compounds influence epigenetic modification of CRC-related genes, but additional studies are needed to understand the mechanisms of action of putative protective nutrients, as most of the evidence comes from in vitro or animal studies.

Nutritional therapies based on epigenetically active nutrients shall likely represent shortly a fruitful research field.

## 9. Conclusions

The latest WCRF/AICR report assesses that CRC primary prevention mainly consists of a healthy diet and a physically active lifestyle. A strong relationship between diet and the development of CRC is widely accepted (Figure 1). Consumption of whole grains, dietary fiber, and dairy products is protective, which is the opposite to the consumption of red and processed meat, and fat-rich diets. Some evidence indicates that consuming foods containing vitamin C, E, and D as well as some minerals (e.g., calcium and selenium) might decrease the risk of CRC [4]. However, studies on incidence and mortality are challenging, due to the sample size and duration of randomized trials required to test the effect of dietary intervention. Moreover, since specific nutrients are not consumed in isolation, but as part of dietary patterns, and dietary components interact with each other, the actual effect of diet on CRC risk may become apparent only when individual components are considered as a whole. Thus, which type of whole diets proves of real benefit is still largely undefined. More so for recommended doses of individual foodstuffs.

Due to the time required for the development of CRC, most hypotheses deriving from in vitro or animal studies, have been tested in humans as polyp prevention trials, in which the colorectal polyps are used as a biomarker of CRC risk. This strategy, however, might not be fully satisfactory.

Despite the complex interplay with the dietary pattern, the microbiome is recognized as central in the development of CRC, but current knowledge is only the tip of the iceberg and dietary recommendations aiming to select or inhibit individual phyla or bacterial strains are presently out of reach.

Epigenetic modifications induced by specific nutrients or microbial metabolites are a new and promising research area that could significantly contribute to unravel the complex colorectal carcinogenetic process.

Current dietary advice is aimed at limiting unhealthy, fat- and protein-rich Western diets, in favor of increased consumption of fruit, vegetables, and cereals, or the so-called Mediterranean diet.

Diet, however, is the expression of complex cultural interactions and is rapidly evolving. There is an important variation in trends of colorectal cancer incidence worldwide which were found to be related to the dietary habits of each country [118]. Nowadays, the so-called Mediterranean diet markedly differs from that of a few decades ago. This is evidenced by the increasingly frequent habit of following a high-calorie, high-fat, almost fibreless diet in most of the urban areas of countries that traditionally followed the Mediterranean diet.

Food is a significant part of the culture of humans and is central to life and well-being. Local dietary habits should always be considered and suggesting just one healthy diet to different populations would likely lead to low adherence and unsatisfactory performance in the prevention of CRC. However, the application of legislative and educational measures promoting a healthy diet has become an urgent issue to stop the increasing tendency of colorectal cancer reported worldwide [118]. Future recommendations shall likely be tailored to individual patients, considering genetic and cultural backgrounds, as well as individual risk factors, the interaction between nutrients and a patient’s specific microbiota.
